# High Prevalence of *Candida auris* Colonization during Protracted Neonatal Unit Outbreak, South Africa

**DOI:** 10.3201/eid2909.230393

**Published:** 2023-09

**Authors:** Liliwe Shuping, Tsidiso G. Maphanga, Serisha D. Naicker, Ruth Mpembe, Nqobile Ngoma, Sithembiso Velaphi, Firdose Nakwa, Jeannette Wadula, Prenika Jaglal, Nelesh P. Govender

**Affiliations:** National Institute for Communicable Diseases, Johannesburg, South Africa (L. Shuping, T.G. Maphanga, S.D. Naicker, R. Mpembe, N. Ngoma, N.P. Govender);; Chris Hani Baragwanath Academic Hospital, Johannesburg (S. Velaphi, F. Nakwa);; University of the Witwatersrand, Johannesburg (S. Velaphi, F. Nakwa, J. Wadula, P. Jaglal, N.P. Govender);; National Health Laboratory Service, Johannesburg (J. Wadula, P. Jaglal);; University of Cape Town, Cape Town, South Africa (N.P. Govender);; St. George’s University of London, London, UK (N.P. Govender);; University of Exeter, Devon, UK (N.P. Govender)

**Keywords:** Candida auris, fungi, candidemia, fungal infections, colonization, neonatal unit, newborn, protracted outbreak, South Africa

## Abstract

One third of patients were colonized by *Candida auris* during a point-prevalence survey in a neonatal unit during an outbreak in South Africa. The sensitivity of a direct PCR for rapid colonization detection was 44% compared with culture. The infection incidence rate decreased by 85% after the survey and implementation of isolation/cohorting.

*Candida auris* has been recognized as a critical priority pathogen globally, causing invasive infections and persistent outbreaks in healthcare facilities (*1*). In June 2019, an outbreak dominated by *C. auris* clade III occurred in a 185-bed neonatal unit of a national central hospital located in Gauteng Province, South Africa. To contain the outbreak, multiple infection prevention and control (IPC) measures were implemented (Appendix), including colonization screening for contact patients housed in the same cubicle as babies who had positive cultures. Despite those measures, sustained control was not achieved, similar to the case for other prolonged outbreaks (*2*,*3*). Although small section-wide colonization point-prevalence surveys (PPS) were conducted earlier for control (Figure 1), a comprehensive unit-wide PPS was never undertaken. We describe a unit-wide PPS conducted before the neonatal unit was relocated to a new facility as part of a longstanding renovation plan.

**Figure 1:**
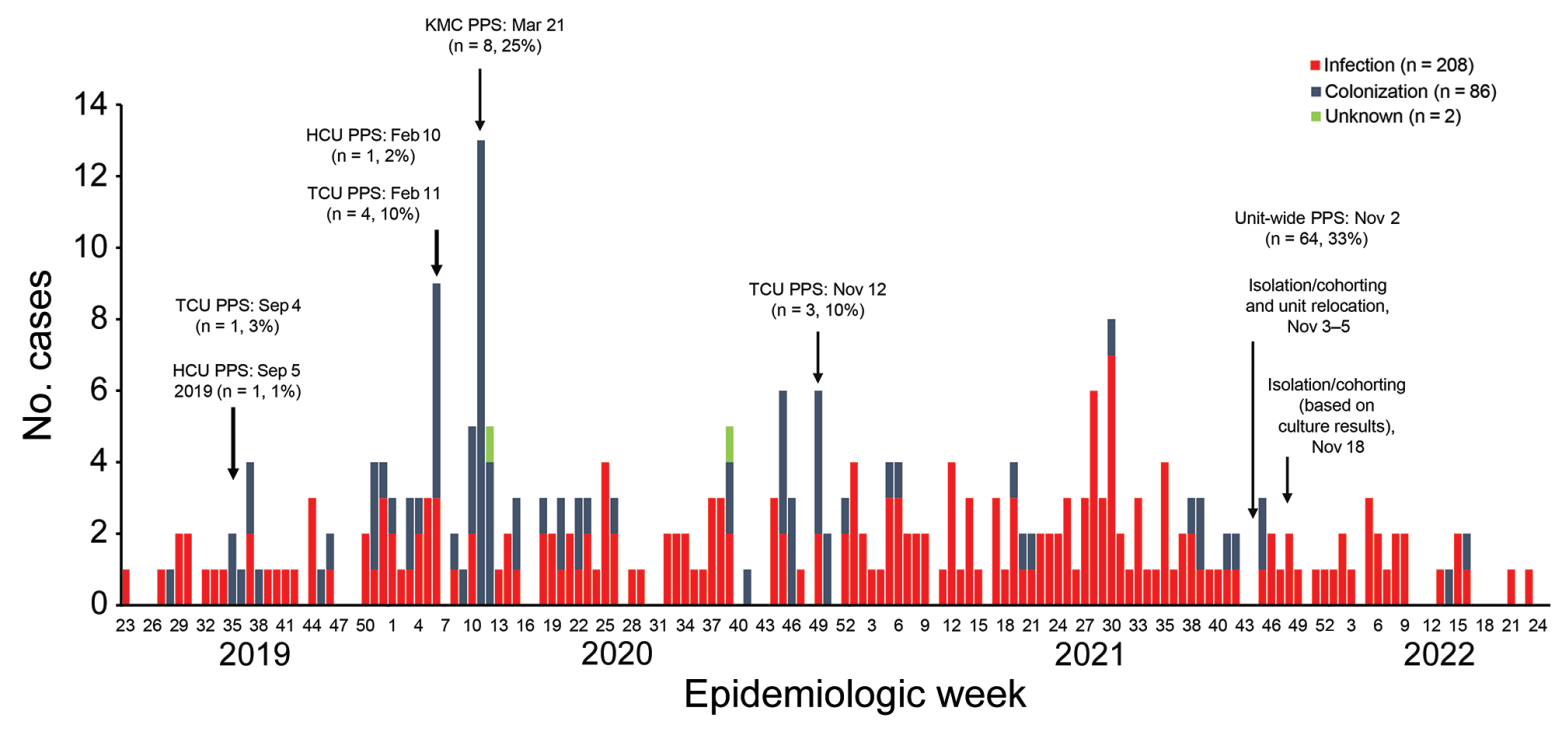
Timeline of culture-confirmed *Candida auris* infection and colonization in neonatal unit, Chris Hani Baragwanath Academic Hospital, Johannesburg, South Africa, June 1, 2019‒June 24, 2022. HCU, high care unit; KMC, kangaroo mother and child care; PPS, point-prevalence survey; TCU, transitional care unit.

## The Study

Institutional ethics approval for public health surveillance and outbreak investigations was granted by the University of the Witwatersrand HREC (Medical) (M210752). Permission to conduct the survey was granted by the hospital’s Medical Advisory Committee, Chief Executive Officer, and the Paediatric Department management.

The aim of the PPS was to reduce *C. auris* transmission in the new facility. The PPS was conducted on November 2, 2021 (3 days before the relocation), to establish colonization status and implement cohorting/isolation for affected infants. We used a direct reverse transcription PCR (RT-PCR)‒based method for rapid detection and compared this to culture as the reference standard. We collected composite skin swab specimens from the axilla and groin (*4*) and used selective and enrichment methods to isolate *C. auris* in culture (Appendix). We used the one-step SYBR PrimeScript RT-PCR Kit II (TaKaRa Bio, Inc., https://www.takarabio.com), according to Sexton et al. (*5*).

We swabbed 195 infants; RT-PCR results for 55 (93%) of 59 infants admitted to the neonatal intensive care unit and transitional care unit were available within 24 hours of specimen collection. Samples from those sections were prioritized because of high previous number of infections (Appendix Figure 1). Processing of the remaining swab samples was completed within 48 hours. The prevalence of *C. auris* colonization by RT-PCR was 15% (29/195) (Table 1). All culture results were available within 17 days after specimen collection because of multiple processing steps (Appendix Figure 2). With culture, the prevalence of *C. auris* was 32% (63/195). The overall prevalence was 33% (64/195). The sensitivity of the RT-PCR compared with culture was 44% (95% CI, 32%–58%). The sensitivity was highest in the high-care surgical unit and the neonatal intensive care unit, where the prevalence of colonization was highest on the day of the unit-wide PPS (Table 2).

**Table 1:** Prevalence of *Candida auris* colonization by direct SYBR PrimeScript RT-PCR and selective/enrichment culture with MALDI-TOF mass spectrometry identification in neonatal unit of Chris Hani Baragwanath Academic Hospital, Johannesburg, South Africa, November 2, 2021*

Neonatal unit	No. swabbed	Prevalence by RT-PCR	Prevalence by culture	Overall prevalence
Intensive care	12	6 (50)	10 (83)	10 (83)
Transitional care	46	8 (17)	14 (30)	14 (30)
High care surgical	10	5 (50)	6 (60)	7 (70)
High care	97	7 (7)	27 (28)	27 (28)
Kangaroo mother and child care	30	3 (10)	6 (20)	6 (20)
Total	195	29 (15)†	63 (32)	64 (33)

*Values are no. (%) except as indicated. MALDI-TOF, matrix-assisted laser desorption/ionization time-of-flight; RT-PCR, reverse transcription PCR. †One infant had insufficient sample for PCR; however, this infant was included in the denominator when calculating prevalence.

**Table 2:** Diagnostic accuracy measures for a direct SYBR PrimeScript RT-PCR compared with culture (standard) during a *Candida auris* colonization survey in neonatal unit of Chris Hani Baragwanath Academic Hospital, Johannesburg, South Africa, November 2, 2021*

Neonatal unit	Sensitivity	Specificity	Positive predictive value	Negative predictive value	Diagnostic accuracy
Intensive care	60 (26–88)	100 (16–100)	100 (54–100)	33 (4.0–78)	67 (35–90)
Transitional care	57 (29–82)	100 (89–100)	100 (63–100)	84 (69–94)	87 (74–95)
High care surgical	67 (22–96)	75 (19–99)	80 (28–9)	60 (15–95)	70 (35–93)
High care	26 (11–46)	100 (95–100)	100 (59–93)	78 (67–86)	79 (70–87)
Kangaroo mother and child care	50 (12–88)	100 (86–100)	100 (29–100)	89 (71–98)	90 (73–98)
Overall	44 (32–58)	99 (96–100)	97 (82–100)	79 (72–85)	81 (74–85)

*Values in parentheses are 95% CIs. One patient had a positive RT-PCR result but a negative culture. A heavy growth of *Staphylococcus aureus* from this patient’s sample could have overgrown *C. auris*. RT-PCR, reverse transcription PCR.

All infants who were colonized with *C. auris* were immediately placed in isolation/cohorted in a separate section with contact precautions after either a positive PCR result or culture result. Infants who were positive for *C. auris* based on PPS results or who had a previous culture-positive diagnostic specimen for *C. auris* were not transferred to the new facility. Instead, they remained in the isolation/cohorting section of the old neonatal unit until discharge. Because swab specimen culture results were still unknown on the relocation day, admitted PCR-negative and subsequently admitted infants were housed in separate wings in the new unit. Apart from that measure and the allocation of dirty and clean equipment areas, IPC practices in the new unit remained largely unchanged.

**Figure 2:**
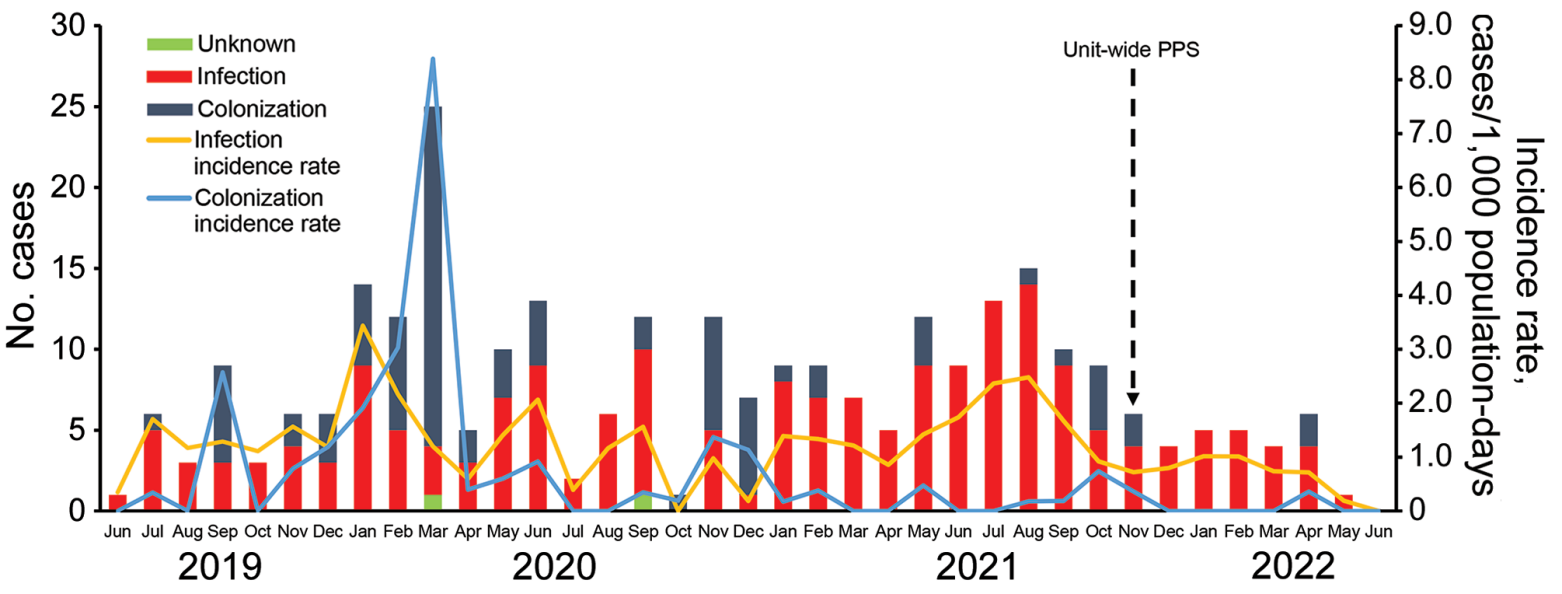
Timeline of new cases and incidence rate of culture-confirmed *Candida auris* infection (n = 194) and colonization (n = 86) in neonatal unit, Chris Hani Baragwanath Academic Hospital, Johannesburg, South Africa, June 1, 2019‒June 24, 2022. PPS, point-prevalence survey.

Using archived laboratory data, we analyzed incidence rates of *C. auris* infection (isolation from normally sterile specimens) or colonization (isolation from nonsterile specimens) in the unit before the PPS (January 1, 2019–November 2, 2021) and after the PPS and relocation (November 3, 2021–June 24, 2022) (Figure 2). Before the PPS, 167 new cases of *C. auris* infection were diagnosed, an incidence rate of 1.3 cases/1,000 patient-days. After the survey, 27 new cases of infection were diagnosed, an 85% decrease in the infection incidence rate to 0.2 cases/1,000 patient-days after PPS. The incidence rate of *C. auris* colonization was 0.6 cases/1,000 patient-days (n = 82) before the PPS and 0.1 cases/1,000 patient-days after (n = 4).

## Conclusions

Compared with previous limited surveys in the unit, we determined a high prevalence of *C. auris* colonization during the unit-wide PPS, probably a major factor in ongoing transmission within the neonatal unit (*6**,**7*). Screening of direct contacts and surveys limited to specific sections of the unit probably missed colonized patients in other areas, and our results emphasize the need for routine unit-wide surveys, which are more effective in detecting the true extent of colonization during protracted *C. auris* outbreaks.

In the months after the unitwide PPS, infection and colonization incidence decreased. However, infections and colonization (albeit to a lesser extent) continued to occur. Assuming that skin colonization always precedes invasive infection, the continued occurrence of *C. auris* infections suggests the PPS was only partially successful at control. Culture-based methods used for identification delayed implementation of contact precautions because of a long turnaround time. The RT-PCR intended for rapid identification of colonization had a lower sensitivity than the >90% reported previously (*5*). The low observed sensitivity was possibly caused by low fungal load in the swab specimens, supported by a longer time-to-culture-positivity for PCR-negative/culture-positive swab specimens than for PCR-positive/culture-positive swab specimens (Appendix Table 1). In addition, a higher fungal burden on patient skin in high-prevalence neonatal unit sections might have improved detection (*7*). Nonetheless, we could not exclude PCR inhibitors as a reason for low sensitivity because our assay lacked an internal control.

Despite the limitations of our case detection methods during the PPS, the substantial decrease in infection incidence strongly suggests that the PPS and related IPC measures played a crucial role in control. Although colonization incidence also decreased after the PPS, we are uncertain whether that was a real decrease. The incidence in the period before the PPS included colonized patients identified during limited surveys, resulting in more colonization cases potentially being detected in that period compared with the post-PPS period.

Undetected colonization and persisting IPC challenges, such as staff shortages and bed occupancy in excess of capacity, all probably contributed to the continued transmission within the unit. Topical chlorhexidine gluconate or terbinafine could lead to skin decolonization (*8**,**9*). However, determining the optimal skin concentration, required contact time, and number of applications for sustained *C. auris* clearance and ensuring safety in neonatal populations remain unresolved (*10*). A comprehensive bundle of IPC measures, which includes routine PPS to assess skin colonization, preferably using a more sensitive PCR method (such as TaqMan chemistry) (*7**,**11*), along regular audits of adherence to contact precautions, surgical aseptic technique, device care protocols, and periodic environmental sampling to guide cleaning and decontamination efforts, should be implemented. This system could be challenging and costly to maintain in a large unit; however, these measures are crucial for control. In conclusion, regular PPS should be conducted in neonatal units experiencing ongoing *C. auris* outbreaks to identify colonized persons and implement IPC precautions to prevent spread.

Appendix Additional information on high prevalence of *Candida auris* colonization during protracted neonatal unit outbreak, South Africa.
