# Supplementary material for: High Prevalence of Candida auris Colonization during Protracted Neonatal Unit Outbreak, South Africa
Source: Emerg Infect Dis. 2023 Sep;29(9):1913–6. doi: 10.3201/eid2909.230393 (PMC10461653; doi:10.3201/eid2909.230393)
Supplement: Appendix — Additional information on high prevalence of Candida auris colonization during protracted neonatal unit outbreak, South Africa. [file 23-0393-Techapp-s1.pdf]

Article DOI: https://doi.org/10.3201/eid2909.230393

EID cannot ensure accessibility for Supplemental Materials supplied by authors. Readers who have difficulty accessing supplementary content should contact the authors for assistance.

High Prevalence of *Candida auris* Colonization during Protracted Neonatal Unit Outbreak, South Africa

Appendix

Methods

Setting

The neonatal unit of the Chris Hani Baragwanath Academic Hospital comprised several sub-sections, including the 18-bed neonatal intensive care (NICU), 42-bed transitional care unit (TCU), 6-bed high care surgical unit (HCSU), 105-bed standard high care unit (HCU) and 14-bed kangaroo mother and child care (KMC) unit. Babies admitted to the NICU and HCSU (level 3 nurseries) were those critically ill, requiring invasive mechanical ventilation and inotropic support. Critically ill neonates (<900 g), newborn babies with birthweight <1200 g regardless of severity of illness, those requiring TPN, those with central lines, and those with asphyxia on therapeutic hypothermia were admitted to the TCU (level 2 nursery). Newborn infants weighing 1,200–1,700 g irrespective of severity of illness and/or those requiring supplemental oxygen, antibiotics or on rehabilitation were admitted to the HCU (level 1 nursery). Babies awaiting weight gain (>1700 g) were admitted to the KMC (level 1 nursery).

Control Measures

Throughout the outbreak, control measures were implemented in the unit, including placement in isolation/cohorting of babies with positive specimens, discontinuing fluconazole prophylaxis, a switch to decontamination of surfaces and equipment with sporicidal hydrogen peroxide wipes (when these were available), and staff education among others. Colonization screening targeting high-risk contacts (admitted in same section with a culture-confirmed case) was performed on an ongoing basis from the onset of the outbreak. Although unit-wide point

prevalence surveys (PPS) were not done, small surveys limited to selected sections were performed based on the number of patients with *C. auris* clinical disease. Section-wide PPS were conducted on September 4, 2019 in the TCU (1/30 positive), September 5, 2019 in the HCU (1/79 positive), February 10, 2020 in the HCU (1/64 positive), February 11, 2020 in the TCU (4/40 positive), March 21, 2020 in the KMC (8/32 positive) and on November 12, 2020 in TCU (3/29 positive). Small PPS in the unit were discontinued at the end of 2020. Infants found to be infected or colonized were isolated/cohorted in a separate section of the neonatal unit with dedicated nursing staff and equipment. Wearing of gloves and aprons when treating patients in the isolation section was enforced. Alcohol hand rub and sterile gloves were placed at every infant's bed and sporicidal wipes were used to clean cots and incubators when babies were housed in them. Upon discharge, chlorine-based disinfectants were used for cleaning of equipment when sporicidal wipes were not available. Infants in isolation were never de-isolated until discharge.

Unit-Wide PPS

All infants admitted to the neonatal unit sections on November 2, 2021 during the night shift (between 5:00 a.m. to 7:00 a.m.) were swabbed. Composite moistened skin swabs were collected from the axilla and groin following U.S. Centers for Disease Control and Prevention (CDC) recommendations (*1*). The Amies swabs (Copan Diagnostics, USA) in liquid transport media were immediately transported to the National Institute for Communicable Diseases (NICD) Mycology Reference Laboratory for processing.

Culture

Upon receipt at the reference laboratory, the Amies swabs were incubated at 40°C in a shaking incubator overnight. The swabs were vortexed and 100 µL of the suspension was added to 2 mL of Salt Sabouraud Dulcitol (SSD) broth the following day. Tubes were incubated at 40°C in a shaking incubator for a minimum of 48 hours and a maximum of 7 days with daily monitoring for growth. When turbid, a loopful (≈10 µL) of broth was immediately inoculated onto CHROMagar *Candida* (Becton Dickinson, San Jose, CA) and incubated at 35°C for 72 hours or longer if no growth. On the seventh day, all broth was inoculated onto CHROMagar *Candida* and incubated as above regardless of turbidity. Off-white/pink/purple colonies on chromogenic agar were sub-cultured onto Sabouraud agar plates and incubated at 35°C for 24–

48 hours. The matrix-assisted laser desorption ionization–time of flight mass spectrometry (MALDI-TOF MS) formic acid extraction tube method (Microflex LT; Bruker, Bremen, Germany) was used to identify isolates.

PCR

DNA was extracted from 200 µl of the Amies swab liquid medium following overnight incubation by using the automated Qiagen QIAcube HT extractor with the QIAamp 96 DNA QIAcube HT kit (Qiagen, Germany). The one-step SYBR PrimeScript reverse transcription-PCR (RT-PCR) kit II (TaKaRa Bio, Inc, CA, USA) was used for PCR according to Sexton et al. (*2*). PCR processing was performed in 2 batches, prioritizing samples from the NICU and TCU where most of the infants with *C. auris* infection or colonization had been diagnosed. Each PCR run included a negative extraction control (reagents only), a no-template control (nuclease-free water), and a positive control (DNA extracted from a cultured *C. auris* isolate). No internal amplification control was included in the PCR; however, an inhibitor removal buffer was included during DNA extraction. The diagnostic accuracy estimates and corresponding 95% confidence intervals (CI) for the direct RT-PCR assay compared with culture were computed by using R software (*3*).

Analysis of Culture-Confirmed Cases of *C. auris*

Archived microbiology data from the on-site National Health Laboratory Service diagnostic laboratory were obtained by the NICD. All test results which recorded that *C. auris* had been isolated in culture from January 1, 2019 through June 24, 2022 in the neonatal unit were obtained. This period was selected because the index case related to this outbreak was diagnosed in June 2019. Sporadic neonatal cases of *C. auris* infections had been diagnosed in 2017 but no further cases were identified in 2018. For this analysis, a case of culture-confirmed *C. auris* infection was defined as *C. auris* isolated from blood or cerebrospinal fluid (CSF). Colonization was defined as *C. auris* isolated from specimens other than blood and CSF, including but not limited to, skin swabs, wound/ abscess swabs, intravascular catheter tips and urine. Colonization cases included persons identified through 1) limited PPS conducted in specific sections of the unit, 2) screening of contacts linked to confirmed infection cases, and 3) non-sterile specimens sent for routine diagnostic testing. Colonization cases identified during the current unit-wide PPS were not included when calculating colonization incidence. Additional

infection or colonization episodes in the same infant 30 days after the previous episode were considered recurrent and included. When calculating the incidence rate of *C. auris,* only incident episodes of infection or colonization were included as part of the numerator.

Results

Cases of Culture-Confirmed *C. auris* Infection and Colonization

Over the analysis period, there were 208 cases (including recurrent cases) of culture-confirmed *C. auris* infection of which 51% (102/199) were female with a median age of 21 days (interquartile range [IQR], 14–36). A total of 86 cases of colonization were identified with a median age of 22 days (IQR, 15–37) and 49% (38/77) female. Specimen type was unknown for 2 patients with positive cultures (Figure 1). Patients admitted to the NICU (44%, n = 129) and TCU (37%, n = 110) accounted for most infection and colonization cases (Appendix Figure 1A and 1B). The smallest number of cases were diagnosed in the KMC unit, and all these were cases of colonization (n = 8).

The incidence rate of new *C. auris* infection and colonization was 1.6 cases per 1000 patient-days (monthly range, 0.2 – 9.6) (Figure 2). The incidence rate of infection alone was 1.1 cases per 1000 patient-days with monthly variations for the entire analysis period (range, 0–3.4). The overall colonization incidence rate (excluding babies in the current PPS) was 0.5 cases per 1000 patient-days (range, 0–8.4) and was highest during the months when smaller surveys were conducted in the unit (2.6–8.4 cases per 1000 patient-days) (Figures 1 and 2). In the 6 months following the survey, 27 new cases of invasive infection and 4 new cases of colonization were diagnosed. Three of the 27 new infection cases were screened during the unit-wide PPS and one had a positive colonization swab. Only one of the four new cases of colonization had been screened in the PPS, and they had a positive colonization swab.

References

1. Center for Disease Control and Prevention National Center for Emerging and Zoonotic Infectious Diseases (NCEZID). Division of Foodborne, Waterborne and ED (DFWED). Screening for *Candida auris* Colonization. Fungal Diseases. 2020 [cited 2022 Jun 8]. https://www.cdc.gov/fungal/candida-auris/c-auris-screening.html

2. Sexton DJ, Kordalewska M, Bentz ML, Welsh RM, Perlin DS, Litvintseva AP. Direct detection of emergent fungal pathogen *Candida auris* in clinical skin swabs by SYBR green-based quantitative PCR assay. J Clin Microbiol. 2018;56:1–6. PubMed https://doi.org/10.1128/JCM.01337-18

3. R core Team. R: a Language and Environment for Statistical Computing. R Foundation for Statistical Computing. 2020. Vienna, Austria. https://www.r-project.org

Appendix Table. Time to *C. auris* MALDI-TOF MS ID by RT-PCR and culture results, N = 195

RT-PCR	Culture	No.	No. days from specimen collection to culture/MALDI ID (no., %)
Negative	Positive	35	7 (n = 6, 17%)
			10 (n = 10, 28%)
			14 (n = 19, 54%)
Positive	Positive	28	7 (n = 14, 50%)
			10 (n = 5, 18%)
			14 n = (9, 32%)
Negative (including /1 insufficient)	Negative	131	Not available
Positive	Negative	1	*Staphylococcus aureus* overgrowth

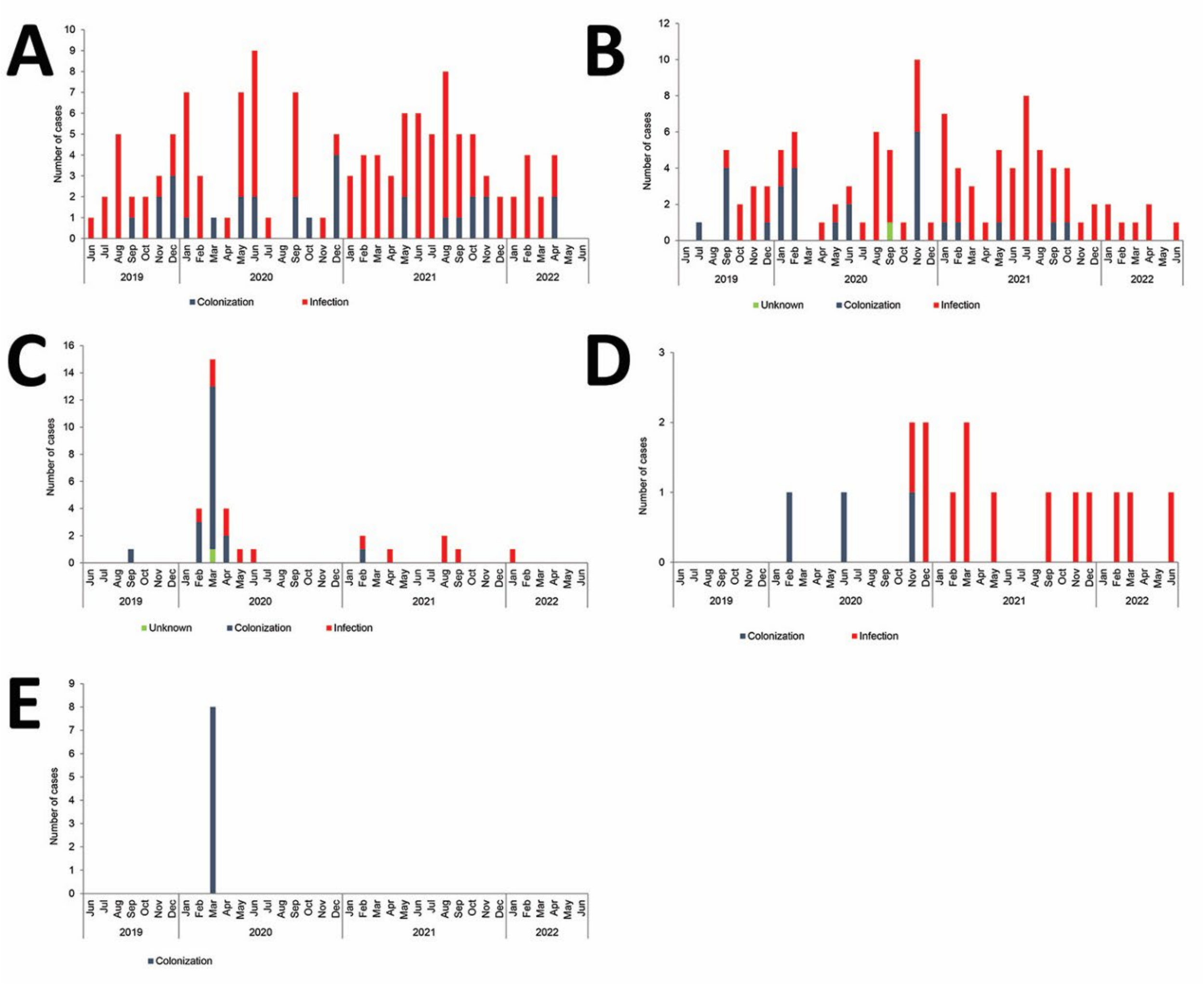

Appendix Figure 1. Cases of culture-confirmed *Candida auris* infection and colonization in the neonatal unit by section. A) Neonatal Intensive Care Unit (n = 129); B) Transitional Care Unit (n = 110); C) High Care Unit (n = 33); D) High Care Surgical Unit (n = 16); E) Kangaroo Mother and Child Care Unit (n = 8), Chris Hani Baragwanath Academic Hospital, June 1, 2019 through June 24, 2022

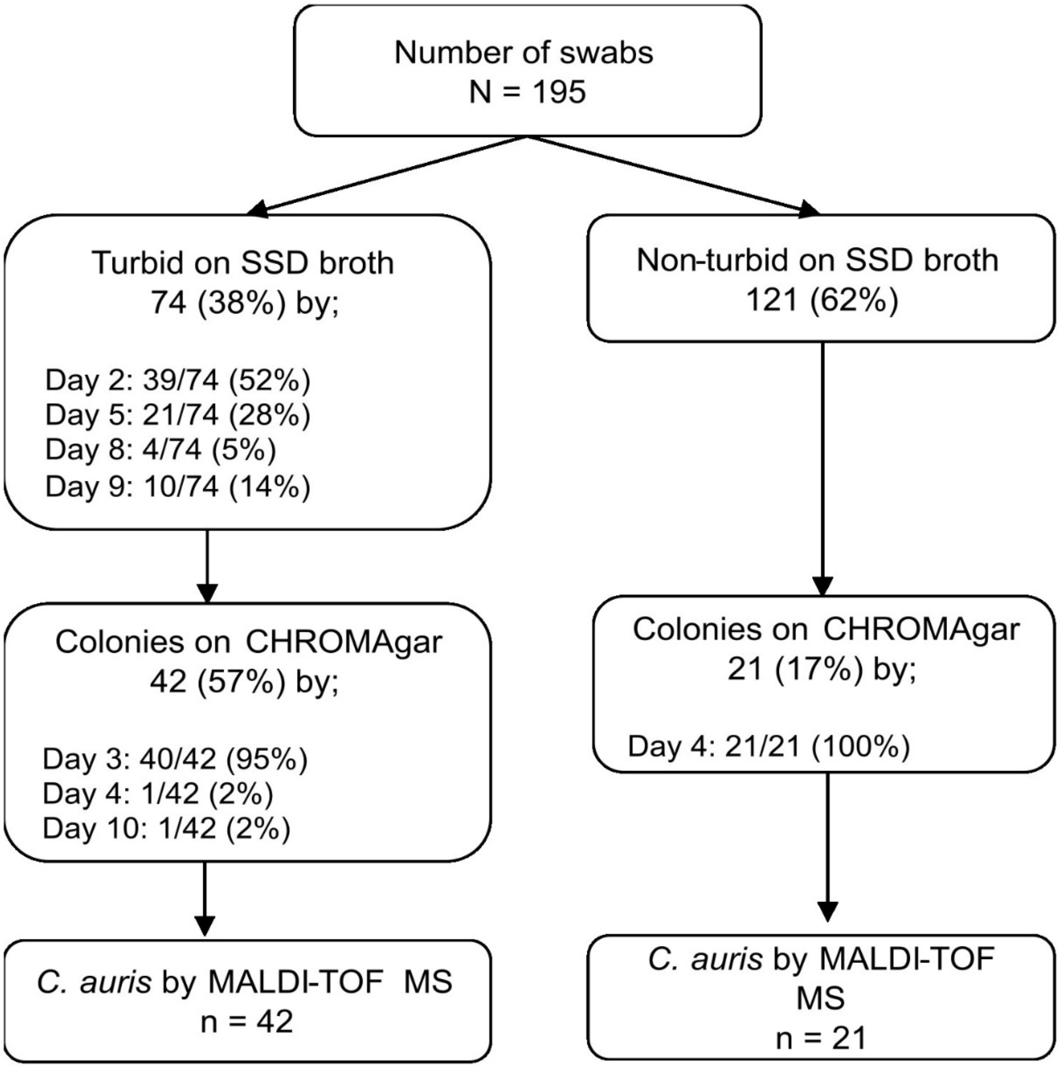

Appendix Figure 2. Time to turbidity of swab samples in Salt Sabouraud Dulcitol (SSD) broth following inoculation and time to growth on CHROMagar after incubation.
